# Sensitive Detection and Monitoring of Senescence-Associated Secretory Phenotype by SASP-RAP Assay

**DOI:** 10.1371/journal.pone.0052305

**Published:** 2012-12-18

**Authors:** Liubao Gu, Masanori Kitamura

**Affiliations:** 1 Department of Molecular Signaling, Interdisciplinary Graduate School of Medicine and Engineering, University of Yamanashi, Chuo, Yamanashi, Japan; 2 Diabetes Care and Research Center, Jiangsu Province Institute of Geriatrics, Nanjing, People’s Republic of China; Boston University Medical School, United States of America

## Abstract

Senescence-associated secretory phenotype (SASP) is characterized by abundant secretion of various proteins in senescent cells and implicated in tumor progression and inflammatory responses. However, the profile of secreted proteins in SASP is different from cell type to cell type, and currently, universal markers for SASP have not been reported. In the present investigation, we show that SASP-responsive alkaline phosphatase (SASP-RAP) serves as a sensitive, general and convenient marker for SASP. Etoposide-treated cells exhibited a senescent phenotype characterized by senile morphology, positive staining for senescence-associated β-galactosidase, growth arrest and induction of p53 and p21^WAF1/CIP1^. In SASP-RAP-transfected cells, exposure to etoposide increased secretion of SASP-RAP time-dependently. The kinetics of secretion was closely correlated with that of activation of the p21^WAF1/CIP1^ promoter and the p16^INK4a^ promoter. The enhanced secretion of SASP-RAP by senescence was also observed in cells treated with other senescence inducers such as trichostatin A, doxorubicin and 4-phenylbutylic acid. The induction of SASP-RAP by senescence was similarly observed in natural replicative senescence. To confirm selectivity of the SASP-RAP response, cells were treated with senescence-related and -unrelated stimuli (IL-1β, LPS, TNF-α and TGF-β), and induction of senescence markers and activity of SASP-RAP were evaluated in parallel. Unlike etoposide, senescence-unrelated stimuli did not induce p53 and p21^WAF1/CIP1^, and it was correlated with lack of induction of SASP-RAP. In contrast, senescence-unrelated stimuli up-regulated conventional indicators for SASP, *e.g.,* MMP-3, IL-6 and TIMP, without induction of senescence. SASP-RAP thus serves as a selective, convenient and general marker for detection and monitoring of SASP during cellular senescence.

## Introduction

Cellular senescence is a state of irreversible growth arrest induced by telomere shortening (replicative senescence), oncogene activation and DNA damage (premature senescence) [1]–[2]. Senescence-associated secretory phenotype (SASP) has been identified as a typical feature of senescent cells, which is characterized by increased expression and secretion of cytokines, chemokines, matrix metalloproteinases (MMPs) and other secretory proteins [3]. Generally, cellular senescence is an anti-tumorigenic mechanism via induction of growth arrest [1]–[4] and triggering immune-mediated clearance of pre-malignant cells [5]. However, on the other hand, SASP causes abundant secretion of various bioactive proteins from senescent cells and thereby activates neighboring non-senescent cancer cells, leading to promotion of tumor growth [6]–[7]. SASP is also considered to be a mechanism responsible for chronic inflammation observed during aging [8]. Towards better understanding of senescence-associated pathologies, detection and monitoring of SASP are essential.

SASP is widely observed in a variety of cell types including fibroblasts, endothelial cells and epithelial cells [3]. Usually, it is accompanied by other senescence phenotypes; *e.g.,* morphological features (large, flat and multinucleated), senescence-associated β-galactosidase (SA-β-gal) activity [9] and activation of p53–p21^WAF1/CIP1^ and p16^INK4a^–Rb signaling cascades [2]. However, the profile of secreted proteins in SASP is different from cell type to cell type, or dependent on different cellular contexts. Some SASP-related factors are up-regulated and secreted in some senescent cells but not in other cell types [3]. Furthermore, production of many SASP factors is not specific to senescence. For example, SASP-related cytokines, chemokines and MMPs are also produced in response to inflammatory stimuli [10]–[12]. Because of this reason, an array of indicators (mRNAs and proteins) must be evaluated by expensive, time-wasting assays, *e.g.,* ELISA, RT-PCR and Northern/Western blot analysis, to prove SASP [7]–[13]. To date, selective, universal and convenient assays for SASP have not been established yet.

During the past decades, secreted alkaline phosphatase (SEAP) has been used as a reporter to evaluate activity of certain promoter/enhancer elements. Normally, alkaline phosphatase is not secreted, but the recombinant SEAP originated from placental alkaline phosphatase is efficiently secreted from transfected cells. The level of SEAP activity detected in culture medium is directly proportional to changes in SEAP mRNA and protein [14]–[15]. As a reporter, SEAP has several important advantages over other reporter molecules including luciferase and β-galactosidase. Because preparation of cell lysates is not required, it is possible to monitor activity of certain promoters/enhancers continuously using identical culture cells. The assay of SEAP using culture medium is faster, easier and less expensive than assays for other reporter enzymes. Furthermore, detection of SEAP activity is very sensitive using a chemiluminescent assay [16]. Other important advantage of SEAP is that background signals due to endogenous alkaline phosphatases are nearly absent. It is because, unlike most endogenous alkaline phosphatases, SEAP is extremely heat stable and resistant to L-homoarginine. The activity of endogenous alkaline phosphatases present in samples can be eliminated completely by preheating the sample at 65°C and assaying in the presence of L-homoarginine without affecting SEAP activity [15].

Recently, we found that, in cells stably transfected with a *SEAP* gene under the control of constitutively active promoters, secretion of SEAP was enhanced during the course of senescence. We also found that it is a general phenomenon that is observed in different cell types triggered by different senescence inducers. The kinetics of SEAP secretion was closely correlated with the kinetics of other senescence markers. Furthermore, the specificity of the response of SEAP to senescence was superior to other conventional markers for SASP such as cytokines, chemokines, MMPs and tissue inhibitor of MMPs (TIMP). In the present report, we demonstrate several data that support our conclusion and propose that SASP-responsive alkaline phosphatase (SASP-RAP) serves as a selective, convenient and general marker for detection and monitoring of SASP during cellular senescence.

## Materials and Methods

### Reagents

Etoposide, trichostatin A, and doxorubicin were purchased from Wako Pure Chemical (Tokyo, Japan), and 4-phenylbutyric acid (4-PBA), PD98059, SB203580 and Akti-1/2 were from Calbiochem (San Diego, CA). Human recombinant tumor necrosis factor-α (TNF-α) and human transforming growth factor-β (TGF-β) were obtained from R&D Systems (Minneapolis, MN), and human recombinant interleukin-1β (IL-1β) was from Genzyme (Cambridge, MA). Lipopolysaccharide (LPS) and SP600125 were purchased from Sigma-Aldrich Japan (Tokyo, Japan). Dehydroxymethylepoxyquinomicin (DHMEQ) [17] was kindly provided by Dr. Kazuo Umezawa (Keio University, Tokyo, Japan).

### Cell Culture

NRK-52E rat renal tubular epithelial cells were purchased from American Type Culture Collection (Manassas, VA). Mouse embryonic fibroblasts (MEF) were purchased from RIKEN BRC Cell Bank (Tsukuba, Ibaragi, Japan). Normal human mesangial cells (NHMC) were purchased from TAKARA (Shiga, Japan). In our experiments, NRK-52E cells were mainly used, because these cells are non-transformed, non-tumorigenic normal cells and can be transfected efficiently with exogenous genes. Cells were cultured in Dulbecco’s modified Eagle’s medium/Ham’s F-12 (Gibco-BRL, Gaithersburg, MD) supplemented with 5–10% fetal bovine serum (FBS).

### Transient Transfection

NRK-52E cells were transfected with pSV40-SEAP (pSEAP2-Control; BD Biosciences, Palo Alto, CA) or pCMV-SEAP (Addgene, Cambridge, MA) using electroporation and treated with senescence inducers. pSV40-SEAP and pCMV-SEAP introduce a *SEAP* gene under the control of the simian virus 40 (SV40) promoter/enhancer and the cytomegalovirus (CMV) promoter, respectively. The culture medium was changed to fresh 1% FBS medium 8 h prior to sampling. The 8 h-conditioned media were then collected and subjected to chemiluminescent assay to evaluate SEAP/SASP-RAP activity. NRK-52E cells were also transiently transfected with p21^WAF1/CIP1^-Luc (provide by Dr. Naoko Ohtani; The Cancer Institute of Japanese Foundation for Cancer Research, Tokyo, Japan) [18] and p16^INK4a^-Luc (provide by Dr. Kiyoshi Nose; Showa University School of Pharmaceutical Sciences, Tokyo, Japan) [19]. The p21^WAF1/CIP1^-Luc and p16^INK4a^-Luc introduce a luciferase gene under the control of the p21^WAF1/CIP1^ promoter and the p16^INK4a^ promoter, respectively. MEF and NHMC were also transfected with pSV40-SEAP using GeneJuice (Novagen, Madison, WI). Co-transfection with pEGFP-N1 (Clontech, Palo Alto, CA) encoding enhanced green fluorescent protein (EGFP) was used to evaluate transfection efficiency.

### Stable Transfection

Using electroporation, NRK-52E cells were transfected with pSV40-SEAP, and stably transfected NRK/SV-SEAP cells were established. Constitutive production of SEAP was evaluated by chemiluminescent assay using culture media, as described before [20]. For time-lapse experiments, the established cells were exposed to etoposide for up to 7 days to induce senescence. Eight hours prior to individual sampling, culture media were replaced with 1% FBS, and the 8 h-conditioned media were subjected to chemiluminescent assay.

### SEAP (SASP-RAP) Assay

Activity of SEAP (SASP-RAP) in culture media was evaluated by a chemiluminescent method using Great EscAPe SEAP detection Kit (BD Biosciences), as described previously [20]. In brief, 5 µl of conditioned media (1% FBS containing medium) was mixed with 15 µl of 1× dilution buffer and incubated for 30 min at 65°C. After the incubation, the samples were mixed with 20 µl of assay buffer containing L-homoarginine, left at room temperature for 5 min and added with 20 µl of chemiluminescent enhancer containing 1.25 mM CSPD chemiluminescent substrate. After incubation in dark for 30 min, the samples were subjected to chemiluminescent assay using a luminometer (Gene Light 55, Microtech Nition, Chiba, Japan). Assays were performed in quadruplicate.

### SA-β-gal Assay

SA-β-gal activity was evaluated as described by Dimri *et al.* [9]. In brief, cells were fixed with 3% formaldehyde for 4 min and incubated at 37°C overnight in 5-bromo-4-chloro-3-indolyl-D-galactopyranoside (X-gal) solution containing 1 mg/ml X-gal (Sigma-Aldrich Japan), 40 mM citric acid/sodium phosphate (pH 6.0), 5 mM potassium ferricyanide, 5 mM potassium ferrocyanide, 150 mM NaCl and 2 mM MgCl_2_. Assays were performed in quadruplicate.

### Northern Blot Analysis

Total RNA was extracted by a single-step method, and Northern blot analysis was performed as described before [21]. cDNAs for SEAP (BD Biosciences), p21^WAF1/CIP1^ (provided from Dr. Konrad Huppi; National Institutes of Health, Bethesda, MD) [22], IL-6 [23], MMP-3 [24] and TIMP-1 [25] were used to prepare radio-labeled probes. Glyceraldehyde-3-phosphate dehydrogenase (GAPDH) and 28S ribosomal RNA were used as loading controls.

### Western Blot Analysis

Western blot analysis was performed as described before [26]. The level of p53 was assessed using anti-p53 antibody (Cell Signaling, Beverly, MA). The level of β-actin was assessed using anti-β-actin antibody (Sigma Aldrich Japan) as a loading control. Blots were visualized using the enhanced chemiluminescent system (Amersham Biosciences, Buckinghamshire, UK).

### Luciferase Assay

Activity of luciferase was evaluated by Luciferase Assay System (Promega; Madison, WI) according to the manufacturer’s protocol [27]. Assays were performed in quadruplicate.

### Formazan Assay

The number of viable cells was assessed by formazan assay using Cell Counting Kit-8 (Dojindo Laboratory; Kumamoto, Japan) [28]. Assays were performed in quadruplicate.

### Statistical Analysis

Data were expressed as means ± SE. Statistical analysis was performed using the non-parametric Mann-Whitney *U* test to compare data in different groups. A *p* value <0.05 was considered to indicate a statistically significant difference.

## Results

### Induction of Senescence by Etoposide

**Figure 1:**
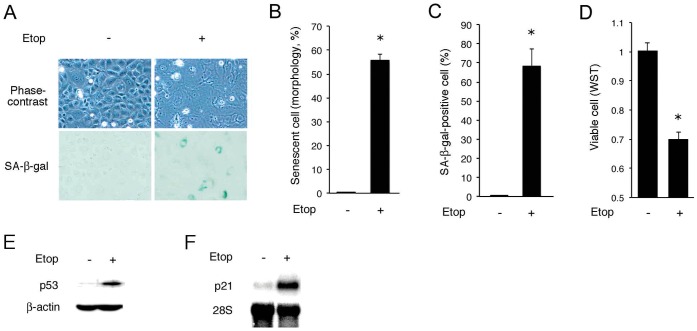
Induction of senescence by etoposide. (A) NRK-52E cells were treated with 1 µg/ml etoposide (Etop) for 3 days and subjected to phase-contrast microscopy and SA-β-gal staining. (B–D) Quantitative assessment of senescent morphology (B), positive staining for SA-β-gal (C) and viable cell number estimated by formazan (WST) assay (D) in etoposide-treated cells. Assays were performed in quadruplicate. Data are expressed as means ± SE, and asterisks indicate statistically significant differences (*p*<0.05). (E, F) NRK-52E cells were exposed to etoposide for 12 h and subjected to Western blot analysis of p53 (E) and Northern blot analysis of p21^WAF1/CIP1^ (F). Levels of β-actin and 28S ribosomal RNA are shown at the bottom as loading controls.

Previous reports demonstrated that low concentrations of etoposide, a genotoxic agent, induced senescent morphology in fibroblasts [29]. To examine whether etoposide induces senescence in NRK-52E cells, we treated the cells with 1 µg/ml etoposide, and microscopic analysis was performed after 3 days. The etoposide-treated cells exhibited a large, extended and flattened cell shape typical of senescence (Figure 1A, top). This morphological change was associated with positive staining for SA-β-gal (Figure 1A, bottom). Quantitative analysis showed that 60–70% of etoposide-treated cells exhibited a senescent phenotype when assessed by morphological changes (Figure 1B) and SA-β-gal staining (Figure 1C). Although cell death was not significantly induced by 3 day-exposure to etoposide (not shown), the number of viable cells in the etoposide group was less than half of that in the untreated group (Figure 1D), suggesting induction of growth arrest. Etoposide also increased p53 protein level (Figure 1E) and induced expression of p21^WAF1/CIP1^ (Figure 1F), both of which are well-known markers for senescence.

### Response of SEAP/SASP-RAP to Etoposide-induced Senescence

**Figure 2:**
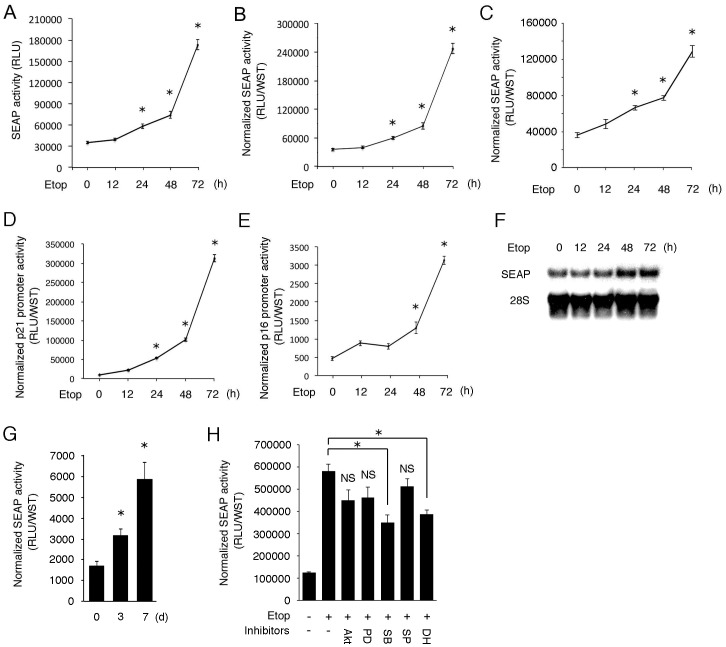
Response of SEAP (SASP-RAP) to etoposide-induced senescence. (A–C) NRK-52E cells were transfected with pSV40-SEAP (A, B) or pCMV-SEAP (C) and treated with etoposide for indicated time periods. Culture medium was changed with fresh 1% FBS, incubated for 8 h before sampling and subjected to chemiluminescent assay (A). SEAP (SASP-RAP) activity was normalized by the level of viable cells estimated by formazan assay, and relative values are shown in (B) and (C). (D, E) Cells transfected with p21^WAF1/CIP1^-Luc (D) or p16^INK4a^-Luc (E) were treated with etoposide for indicated time periods and subjected to luciferase assay and formazan assay. The values were normalized by the level of viable cells. Assays were performed in quadruplicate. Data are expressed as means ± SE, and asterisks indicate statistically significant differences (*p*<0.05). (F, G) NRK-52E cells were stably transfected with a pSV40-SEAP. Established NRK/SV-SEAP cells were treated with etoposide for up to 72 h (F) or 7 days (G) and subjected to Northern blot analysis (F) and chemiluminescent assay (G) of SEAP. (H) Cells were transfected with pSV40-SEAP, treated with etoposide in the presence of indicated inhibitors [Akti-1/2 (Akti), 10 µM; PD98059 (PD), 50 µM; SB203580 (SB), 25 µM; SP600125 (SP), 10 µM; DHMEQ (DH), 5 µg/ml] for 3 days and subjected to chemiluminescent assay. NS, not statistically significant.

In SASP, a wide range of proteins including cytokines/chemokines, growth factors, proteinases and their regulators are secreted abundantly. It indicates that SASP is caused not through activation of particular signaling pathways, but via activation of general machinery responsible for transcription, translation and/or protein secretion. If so, some secretory reporter protein could serve as an indicator for SASP. To examine this possibility, NRK-52E cells were transfected with a SEAP gene under the control of the SV40 promoter and stimulated with etoposide for up to 72 h. Eight hours before sampling, the medium was changed with fresh 1% FBS. After 8 h, culture media and cells were collected and subjected to chemiluminescent assay and formazan assay, respectively. As shown in Figure 2A, secretion of SEAP increased in etoposide-exposed senescent cells in a time-dependent manner. The increase in SEAP activity was not due to altered cell number, because the significant up-regulation of SEAP was observed even when the SEAP activity was normalized by the number of viable cells (Figure 2B). Furthermore, following the exposure to etoposide, a time-dependent increase in SEAP secretion was also observed in cells that produce SEAP under the control of other constitutively active regulatory elements including the CMV promoter (Figure 2C). These results suggest a possibility that SEAP serves as an indicator for SASP.

We compared the kinetics of SEAP activity with that of the senescence biomarkers in etoposide-treated cells. Induction of p21^WAF1/CIP1^ and p16^INK4a^ is known to be a typical phenomenon observed in senescent cells. Therefore, cells were transfected with p21^WAF1/CIP1^-Luc or p16^INK4a^-Luc that introduce a luciferase gene under the control of the p21^WAF1/CIP1^ promoter or the p16^INK4a^ promoter. The cells were then exposed to etoposide for up to 72 h and subjected to luciferase assay. The luciferase activity was normalized by the values of formazan assay. The results showed that the time-dependent increase in SEAP activity was closely correlated with the induction of p21^WAF1/CIP1^ and p16^INK4a^ promoter activity (Figures 2D, 2E).

SASP may be induced by transcriptional activation via particular kinases and transcription factors [13]–[30]. To examine whether the induction of SEAP occurs at the transcriptional level, we established NRK-52E cells constitutively expressing SEAP under the control of the SV40 promoter. When the NRK/SV-SEAP cells were treated with etoposide, the expression of SEAP mRNA was up-regulated in a time-dependent manner (Figure 2F). It was associated with a time-dependent increase in SEAP activity (Figure 2G). These results suggest that, like conventional SASP factors, transcriptional activation is also involved in the up-regulation of SEAP in senescent cells.

Previous reports suggested that, among various kinases and transcription factors, p38 mitogen-activated protein (MAP) kinase and nuclear factor-κB (NF-κB) contribute to the transcriptional induction of SASP factors [13]–[30]. We examined roles of several kinases and NF-κB in the induction of SEAP by etoposide. For this purpose, cells were treated with etoposide in the presence of inhibitors of Akt (Akti-1/2), extracellular signal-regulated kinase (PD98059), p38 MAP kinase (SB203580), c-Jun N-terminal kinase (SP600125) and NF-κB (DHMEQ), and activity of SASP-RAP was evaluated. The result showed that inhibition of p38 MAP kinase or NF-κB, but not other kinases, significantly attenuated induction of SEAP activity in senescent cells (Figure 2H). This result further confirmed the close correlation between expression of SASP factors and induction of SEAP. Based on these results, we use the word “SASP-RAP (SASP-responsive alkaline phosphatase)” instead of “SEAP” in the following studies.

### Response of SASP-RAP to Senescence under Different Cellular Contexts

**Figure 3:**
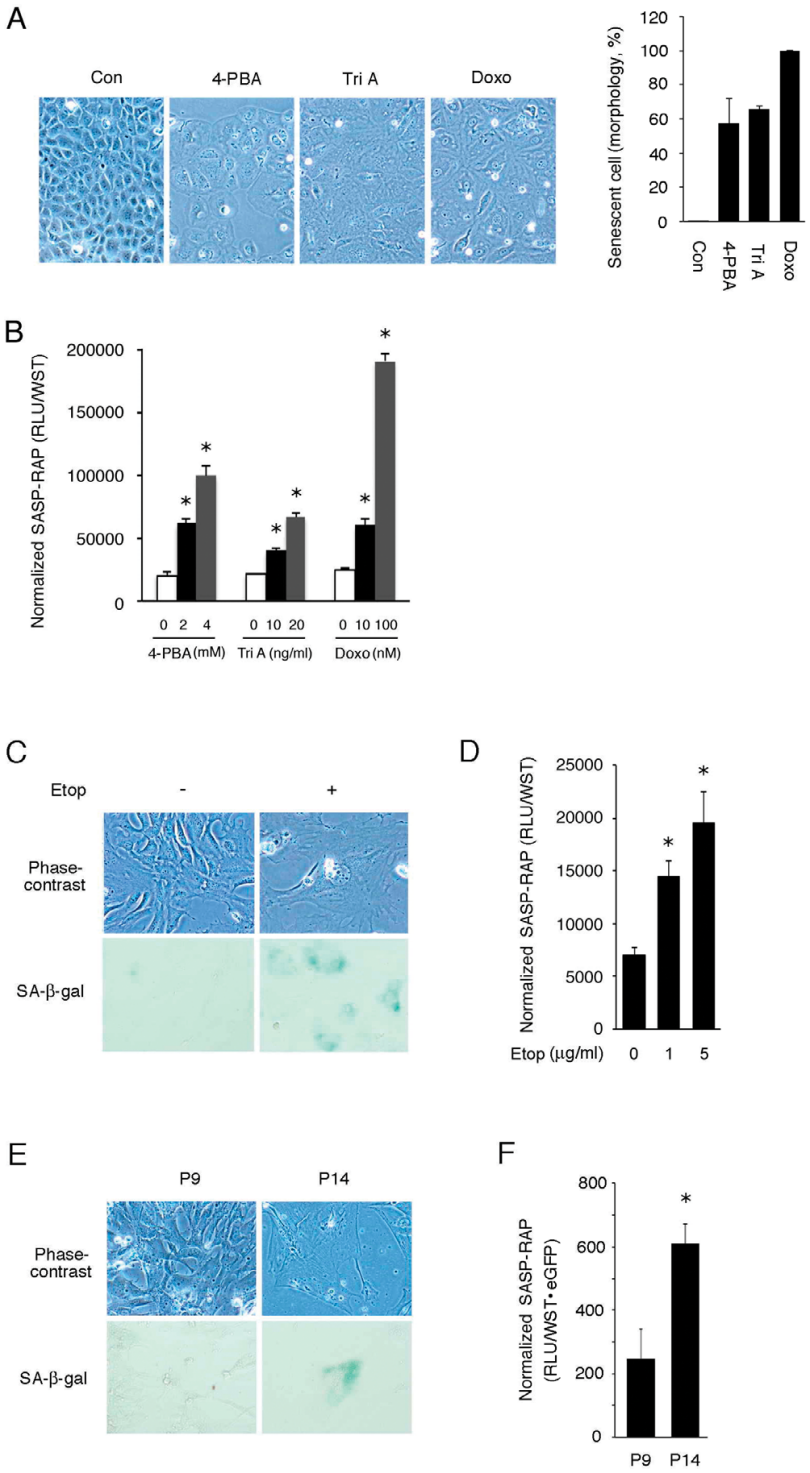
Response of SASP-RAP to senescence under different cellular contexts. (A) NRK-52E cells were treated with 4 mM 4-PBA, 20 ng/ml trichostatin A (Tri A) and 100 nM doxorubicin (Doxo) for 3 days and subjected to phase-contrast microscopy. Quantitative assessment of senescent morphology is shown in the right graph. (B) Cells were transfected with pSV40-SEAP and treated with 4-PBA, trichostatin A or doxorubicin at indicated concentrations for 3 days and subjected to chemiluminescent assay. (C) MEF were treated with 1 µg/ml etoposide for 3 days and subjected to phase-contrast microscopy and SA-β-gal staining. (D) MEF were transfected with pSV40-SEAP and exposed to 1–5 µg/ml etoposide for 3 days, and culture media were subjected to chemiluminescent assay. (E) Normal human mesangial cells (NHMC) at passage 8 (P8) and passage 13 (P13) were co-transfected with pSV40-SEAP and pEGFP-N1, treated with 1 µg/ml etoposide for 3 days and subjected to phase-contrast microscopy and SA-β-gal staining (E) and chemiluminescent assay (F). In (F), SASP-RAP activity was normalized by both transfection efficiency (percentages of EGFP-positive cells) and viable cell number, and the resultant values are shown. In reporter assays, assays were performed in quadruplicate, and data are expressed as means ± SE. Asterisks indicate statistically significant differences (*p*<0.05).

To evaluate the generality of our finding, we next examined responses of SASP-RAP to senescence triggered by other senescence inducers. For this purpose, SASP-RAP-transfected NRK-52E cells were treated with known inducers of senescence; *i.e*., histone deacetylase inhibitors 4-PBA and trichostatin A, and a topoisomerase inhibitor doxorubicin [31]–[32]. As shown in Figure 3A, treatment with 4-PBA, trichostatin A and doxorubicin for 72 h induced senescent morphology. Under these experimental settings, activity of SASP-RAP was significantly up-regulated in the cells treated with individual agents in a dose-dependent manner (Figure 3B).

The induction of SASP-RAP by senescence was not restricted to a particular cell type and was generally observed in different cells. Consistent with the results in NRK-52E cells, treatment of MEF with etoposide for 3 days induced a large, extended and flattened cell shape typical of senescence (Figure 3C, top). It was associated with induction of SA-β-gal (Figure 3C, bottom). All etoposide-treated MEF exhibited this senescent phenotype. When MEF were transfected with SASP-RAP and treated with etoposide, activity of SASP-RAP in culture media was up-regulated dose-dependently (Figure 3D).

Cellular senescence is classified into premature senescence caused by, for example, DNA damage, and replicative senescence caused via telomere shortening. To investigate whether SASP-RAP is also responsive to replicative senescence, natural senescence of NHMC was used as an experimental model. We found that, when NHMC were serially passaged (1∶ 3 passage), the growth was arrested at 14^th^ passage (P14). The growth arrested NHMC exhibited a large, extended and flattened cell shape (Figure 3E top) and positive staining for SA-β-gal (Figure 3E, middle and bottom) typical of senescence. Of note, all P14 cells exhibited the senescent phenotype, which was never observed in P9 cells. We then compared SASP-RAP activity in senescent NHMC (P14) with that in non-senescent cells (P9). P8 and P13 cells were co-transfected with SASP-RAP and EGFP, and seeded in 96-well plates. The 8 h-conditioned media were used for chemiluminescent assay, and the cells were used for; 1) fluorescent microscopy to evaluate transfection efficiency, and 2) formazan assay to assess the viable cell number. The SASP-RAP activity was normalized by both transfection efficiency and viable cell number, and the resultant values were used for comparison. As shown in Figure 3F, SASP-RAP activity significantly increased in replicative senescent cells, when compared with non-senescent NHMC cells.

### Selectivity of SASP-RAP Response to Senescence

**Figure 4:**
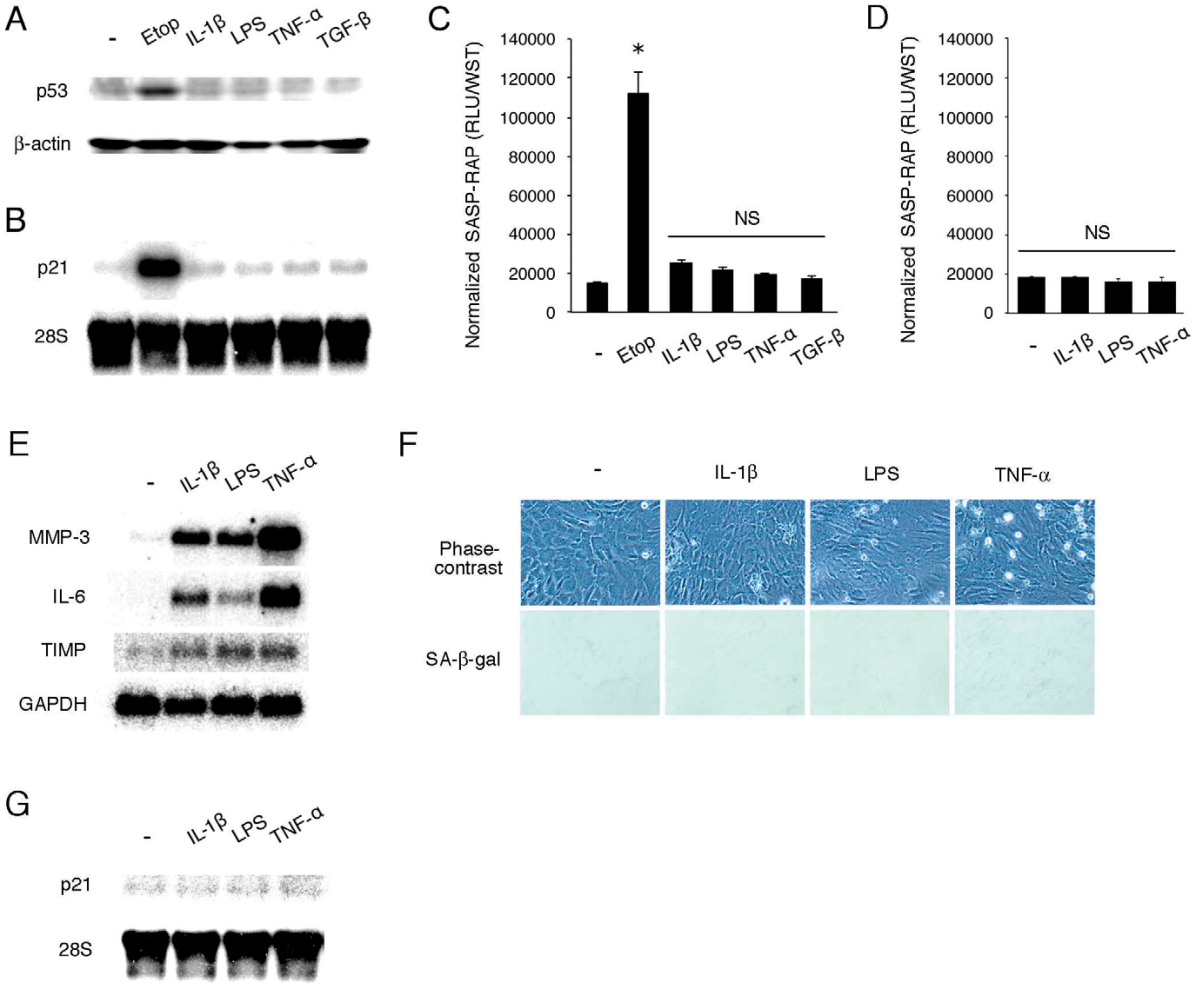
Selectivity of SASP-RAP response to senescence. (A, B) NRK-52E cells were treated with 1 µg/ml etoposide, 1 ng/ml IL-1β, 1 µg/ml LPS, 10 ng/ml TNF-α or 5 ng/ml TGF-β for 3 days and subjected to Western blot analysis for p53 (A) and Northern blot analysis of p21^WAF1/CIP1^ (B). (C, D) NRK-52E cells (C) or MEF (D) were transfected with pSV40-SEAP, treated with indicated agents for 3 days and subjected to SASP-RAP assay. Assays were performed in quadruplicate, and data are expressed as means ± SE. An asterisk indicates a statistically significant difference (*p*<0.05). NS, not statistically significant. (E) MEF were treated with indicated agents for 6 h and subjected to Northern blot analysis for IL-6, MMP-3 and TIMP mRNAs. The level of GAPDH is shown as a loading control. (F, G) MEF were treated with indicated agents for 3 days and subjected to phase-contrast microscopy and SA-β-gal staining (F) and Northern blot analysis of p21^WAF1/CIP1^ (G).

To detect SASP, current approaches use some non-specific, conventional SASP factors, *e.g.*, cytokines, chemokines and MMPs. However, many of these SASP-related factors are up-regulated under situations other than senescence. To confirm selectivity of the SASP-RAP response to senescence, NRK-52E cells were treated with several popular inflammation/tumorigenesis-related stimuli including IL-1β, LPS, TNF-α and TGF-β. As shown in Figures 4A and 4B, etoposide (positive control) induced p53 and p21^WAF1/CIP1^, whereas other stimuli did not induce these senescence markers. When the cells were transfected with SASP-RAP, etoposide induced SASP-RAP activity, whereas none of the senescence-unrelated stimuli triggered up-regulation of SASP-RAP (Figure 4C). The lack of up-regulation was observed not only in NRK-52E cells but also in SASP-RAP-transfected MEF (Figure 4D). On the other hand, conventional indicators for senescence including IL-6, MMP-3 and TIMP were up-regulated by the senescence-unrelated stimuli without induction of senescent morphology, positive staining for SA-β-gal and the senescence marker p21^WAF1/CIP1^ (Figures 4E–G). SASP-RAP thus serves as a marker that is more selective and convenient than conventional indicators for SASP during cellular senescence.

## Discussion

Cellular senescence and its characteristic feature SASP are implicated in various pathophysiological contexts including malignancy, inflammation and aging [4]–[33]. Inhibitors of SASP may be considered as therapeutic agents for a wide range of age-related diseases. Towards better understanding of SASP and development of agents that effectively inhibit SASP-related pathologies, establishment of convenient assays for SASP is essential. Evaluation of various SASP-related molecules is not convenient for this purpose. It is because; (1) the expression/secretion profile of SASP is dependent on cell types or triggers, and an array of SASP factors are often undetectable in certain senescent conditions ([3], and our unpublished observation), (2) production of many SASP factors, especially inflammatory molecules, is not specific to cellular senescence, and (3) conventional evaluation of various SASP factors requires expensive, time-wasting assays. To date, selective, convenient and general assays have not been established for SASP. In the present report, we suggest SASP-RAP as a useful indicator for SASP during cellular senescence. We showed that it is useful to detect SASP in different cell types under different senescent contexts. SASP-RAP can be used for detecting SASP occurred in both telomere-dependent (replicative) and -independent (premature) senescence.

SASP-RAP assay has several advantages over conventional approaches to SASP. First and foremost, in contrast to the current indicators for SASP, SASP-RAP activity is not influenced by senescence-unrelated, non-specific factors such as inflammatory stimuli. Selectivity and specificity of SASP-RAP are thus superior to the conventional markers, as was demonstrated in this report. Second, detection of conventional SASP factors is often difficult in senescent cells, whereas detection of SASP-RAP in transfected cells is easy. Activity of this enzyme can be measured very sensitively and quantitatively by using conventional chemiluminescent systems [34]. The assay is simple and quick, and can be completed within 1–1.5 h [35]. No special instruments or apparatus are needed other than a luminometer. It is contrastive to other conventional approaches, e.g., ELISA, RT-PCR and Northern/Western blot analyses to detect SASP-related cytokines, chemokines and metalloproteinases. The third advantage of the SASP-RAP assay is its low-cost performance. The assay does not require expensive reagents. Small scale assays using 96- or 384-well plates are feasible for evaluation of SASP-RAP. This property allows for economical, high-throughput screening of anti-SASP agents useful for the treatment of cancers and chronic inflammation. The fourth advantage of using SASP-RAP is that, in contrast to other conventional systems, it does not need cell lysates. Only 5 µl of culture medium is sufficient for quantitative assessment of SASP-RAP activity. It means that continuous monitoring of SASP is feasible using serial sampling of conditioned media from identical cell cultures, as shown in this report.

In the SASP-RAP assay, conditioned media are used for evaluation of enzyme activity, and therefore, cells are available for formazan assay. The latter is useful for normalization of SASP-RAP activity to exclude influences of different cell number. However, normalization by formazan assay may underestimate SASP-RAP activity. Based on our experience, SASP-RAP activity was induced by etoposide approximately 7 folds when normalized by values obtained from formazan assay. However, the induction was even higher, approximately 30 times, if SASP-RAP activity was normalized by values obtained from direct cell counting (data not shown). The formazan assay is based upon metabolic reduction of tetrazolium salts to colored formazans by mitochondrial enzymatic systems. Activity of mitochondrial enzymes could be up-regulated in senescent cells with SASP.

Currently, it is unclear how secretion of SASP-RAP is induced by senescence. It may be caused by activation of transcription, enhancement of translation and/or accelerated secretion. In the present investigation, we showed that the up-regulation of SASP-RAP by senescence occurred, at least in part, at the transcriptional level. Furthermore, we also found that p38 MAP kinase and NF-κB partially mediated the induction of SASP-RAP. These findings are consistent with previous reports showing a role of the p38 MAP kinase - NF-κB axis in the transcriptional induction of conventional SASP factors [13]–[30]. However, activation of NF-κB seems to be necessary but not sufficient to induce SASP-RAP, because several NF-κB activators alone did not increase SASP-RAP activity. More general machinery may be involved in the induction of SASP-RAP in senescent cells.

SASP may be induced by chromatin modification, rather than changes in individual transcription factors, because dramatic chromatin alterations occur at senescence [36]–[37]. In senescent cells, disorganization of the nuclear architecture and loss of perinuclear heterochromatin are common findings [38]. Because heterochromatin governs gene silencing and genomic stability, loss of this epigenetic mechanism during senescence may lead to increased transcription of various genes. Moreover, hypermethylation is another general mechanism for transcriptional suppression [39], and during senescence, global DNA methylation is known to decrease in the genome [40]. In senescent cells, therefore, loss of these suppressive mechanisms may lead to a global increase in transcriptional activity and thereby induce a wide range of molecules including SASP-RAP.
